# Single and Transient Ca^2+^ Peaks in Podocytes do not induce Changes in Glomerular Filtration and Perfusion

**DOI:** 10.1038/srep35400

**Published:** 2016-10-19

**Authors:** Sybille Koehler, Sebastian Brähler, Alexander Kuczkowski, Julia Binz, Matthias J. Hackl, Henning Hagmann, Martin Höhne, Merly C. Vogt, Claudia M. Wunderlich, F. Thomas Wunderlich, Frank Schweda, Bernhard Schermer, Thomas Benzing, Paul T. Brinkkoetter

**Affiliations:** 1 Department II of Internal Medicine and Center for Molecular Medicine Cologne, University of Cologne, Cologne, Germany; 2 Department of Pathology & Immunology, Division of Immunobiology, Washington University School of Medicine, St Louis, USA; 3 Cologne Excellence Cluster on Cellular Stress Responses in Aging-Associated Diseases (CECAD), University of Cologne, Cologne, Germany; 4 Systems Biology of Ageing Cologne (Sybacol), University of Cologne, Cologne, Germany; 5 Department of Biological Sciences, Columbia University, New York, NY, USA; 6 Max Planck Institute for Metabolism Research, Cologne, Germany; 7 Department of Physiology, University of Regensburg, Regensburg, Germany

## Abstract

Chronic alterations in calcium (Ca^2+^) signalling in podocytes have been shown to cause proteinuria and progressive glomerular diseases. However, it is unclear whether short Ca^2+^ peaks influence glomerular biology and cause podocyte injury. Here we generated a *DREADD* (Designer Receptor Exclusively Activated by a Designer Drug) knock-in mouse line to manipulate intracellular Ca^2+^ levels. By mating to a podocyte-specific Cre driver we are able to investigate the impact of Ca^2+^ peaks on podocyte biology in living animals. Activation of the engineered G-protein coupled receptor with the synthetic compound clozapine-N-oxide (CNO) evoked a short and transient Ca^2+^ peak in podocytes immediately after CNO administration *in vivo*. Interestingly, this Ca^2+^ peak did neither affect glomerular perfusion nor filtration in the animals. Moreover, no obvious alterations in the glomerular morphology could be observed. Taken together, these *in vivo* findings suggest that chronic alterations and calcium overload rather than an induction of transient Ca^2+^ peaks contribute to podocyte disease.

Diseases of the glomerular filter are a leading cause of end stage renal failure. Podocyte injury is the common final pathway of any glomerular disease. Podocytes are highly specialized epithelial cells and part of the three-layered kidney filtration barrier as they enwrap the glomerular capillaries with their primary and secondary foot processes completely^1^. This barrier consists of the glomerular fenestrated endothelium, the glomerular basement membrane and the podocytes^1^. The latter form an interdigitating pattern of foot processes of adjacent cells. The only cell-cell contact of adjacent podocytes is the slit diaphragm, which is located in between the foot processes^1^. The slit diaphragm does not only serve as a filter to prevent loss of macromolecules from blood into primary urine, but also acts as a signalling platform^2^,^3^,^4^. In the last two decades several genes have been identified to be crucial to mediate signal transduction in podocytes^5^,^6^,^7^,^8^,^9^. In particular, the regulation of the actin-based cytoskeleton via several actin-binding proteins like alpha-actinin-4 and CD2AP^7^,^10^,^11^ appears of specific importance as cytoskeletal changes precede the development of foot process effacement and subsequently the onset of proteinuria as observed in patients with podocyte diseases^12^,^13^,^14^. Calcium was identified as an important second messenger in states of podocyte health and disease^15^. As early as 1978 Kerjaschki *et al*. reported that foot process effacement might be Ca^2+^-dependent^16^. The impact of Ca^2+^ for podocyte biology was further validated in the following years. Overexpression of the G-protein-coupled angiotensin II type 1 receptor, which results in an increased Ca^2+^ flux, causes glomerulosclerosis in rats^17^. Moreover, gain-of-function mutations in the non-selective cation channel TRPC6 were identified to cause familial FSGS^8^,^18^. In addition, TRPC6 was found to be upregulated in different glomerular diseases, e.g. minimal change disease or membranous glomerulonephritis^19^. TRPC channels themselves are tightly regulated^20^ and activated by various stimuli (such as e.g. angiotensin II AT1 receptor). Some reports see a role for TRPC channels to regulate the contractile and motile phenotype of podocytes, as TRPC5 activates Rac1 and TRPC6 RhoA through Ca^2+^ signalling^20^. If this balance is disturbed, podocytes develop either an increased motility through overactivation of Rac1 or a contractile phenotype through overactivation of RhoA. *In vivo*, expression of either constitutively active Rac1 or RhoA both caused albuminuria and foot process effacement^21^,^22^. Additional findings on the Ca^2+^ dependent degradation of the actin-binding protein synaptopodin via the calcineurin/cathepsin-L pathway further emphasise the importance of Ca^2+^ in podocytes^23^,^24^.

Taken together, numerous reports highlight the role of Ca^2+^ dependent signalling in podocytes. However, most studies only provide indirect evidence that indeed Ca^2+^ signalling is required to regulate podocyte function *in viv*o as they focus on e.g. receptor function and do not study Ca^2+^ levels *in vivo*. Due to technical limitations most experiments performed to investigate the role of Ca^2+^ have been performed on cultured podocytes *ex vivo*. To further study the role of Ca^2+^ dependent signalling events in podocytes *in vivo* and its implication on podocyte function we applied the DREADD (Designer Receptor Exclusively Activated by a Designer Drug) concept and generated a novel podocyte-specific transgenic mouse model in which we were able to induce increased intracellular Ca^2+^ levels by administration of a specific chemical compound. To this end, we use a previously well characterized mutant human muscarinic type 3 receptor (hM_3_D), which leads to an exclusive activation by binding of the inert compound Clozapine-N-Oxide (CNO)^25^,^26^. With this *in vivo* approach we demonstrate that single transient Ca^2+^ peaks do not affect glomerular function.

## Results

### Conditional expression of a functional hM_3_D in murine immortalized podocytes

**Figure 1:**
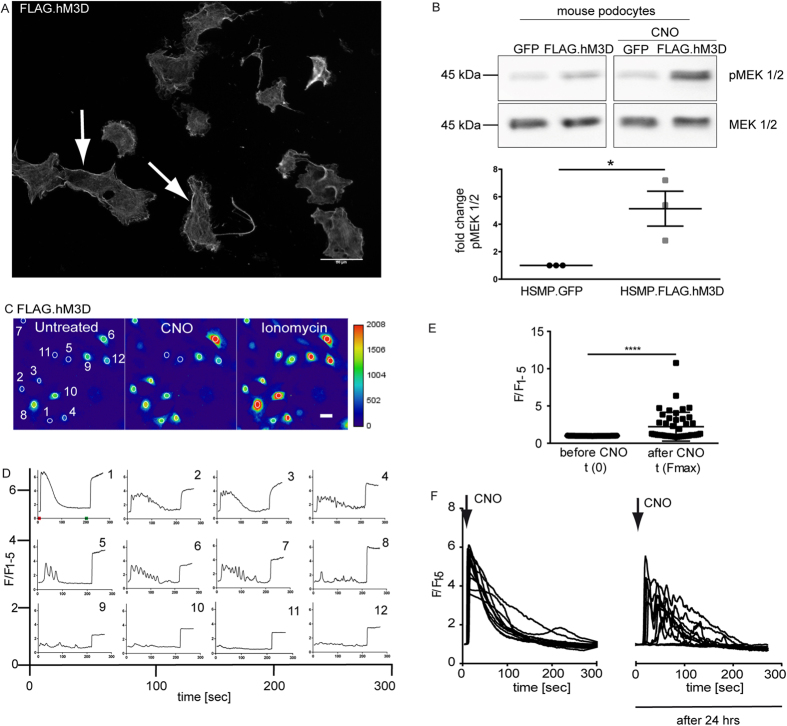
Conditional expression of a functional hM_3_D in murine immortalized podocytes. (**A**) Murine immortalized podocytes stably expressing the FLAG.hM_3_D were stained with a FLAG antibody to confirm expression and membrane localisation (scale bar = 50 μm). (**B**) Immortalized mouse podocytes stably expressing FLAG.hM_3_D or GFP were treated with 1 μM CNO for 10 min. Western Blot analysis revealed a significant increase of the phosphorylation of the MAP-Kinase MEK1/2 after CNO treatment in the receptor expressing cells. (t-test: *p < 0.05). (**C,D**) Representative images of Ca^2+^ imaging with Fluo-8 (scale bar = 20 μm). FLAG hM_3_D expressing podocytes were used for Ca^2+^ imaging with Fluo-8. Administration of 2 μM CNO leads to an immediate increase of intracellular Ca^2+^ levels. The time point of CNO administration is indicated with a red tick in panel one. Ionomycin was used as positive control (indicated with a green tick in panel one). 12 cells are depicted from which some reached saturation of the fluorescence signal. Fluorescence is depicted as F/F_1–5_, where the mean value of the first five measurements (F_1–5_) prior to CNO stimulation was used for normalisation. Experiments were performed in three biological replicates. (**E**) Statistical analysis revealed a significant increase of intracellular Ca^2+^ after CNO stimulation. 45 cells from three biological replicates were included in the statistics. (t-test: p < 0.0001). (**F**) FLAG.hM_3_D expressing podocytes were used for repetitive Ca^2+^ imaging with Fluo-8. Podocytes were treated with CNO at 0 and 24 hrs. At both time points an immediate increase of intracellular Ca^2+^ levels could be observed. Fluorescence is depicted as F/F_1–5_, where the mean value of the first five measurements prior to CNO stimulation was used for normalisation.

We first validated the hM_3_D construct in murine immortalized mouse podocytes and generated cell lines either expressing FLAG-tagged hM_3_D or GFP as control. Immunofluorescence stainings revealed a clear membrane localization of the receptor (Fig. 1A). To verify functionality of the receptor we administered CNO and studied phosphorylation of MAP kinases as a readout for intracellular Ca^2+^ signalling. CNO led to a significantly increased phosphorylation of the MAP-Kinases MEK1/2 (Fig. 1B). In addition, we performed Ca^2+^-imaging studies with the Ca^2+^ indicator dye Fluo-8. Treatment with 2 μM CNO increased intracellular calcium levels in the hM_3_D-expressing cells, but not in control cells (Fig. 1C,D). Depicted are the Ca^2+^ signals of 12 different hM_3_D-expressing cells (Fig. 1D). Of note, some cells showed a single peak upon stimulation with CNO (Fig. 1D upper panel) while others revealed an oscillating response only (Fig. 1D middle panel) or did not react at all (Fig. 1D lower panel). This is most likely a technical artefact due to the lentiviral system used to express the hM_3_D transgene in the podocyte cell line. While the use of this retroviral gene transfer system allows expression of transgenes even in non-proliferating podocytes, which are hardly to transfect, it does not allow to control for expression levels as integration is random and can occur only once or several times. Experiments were performed in three biological replicates and a total of 45 cells were included in the statistical analysis shown in Fig. 1E. We analysed the fluorescence signal after CNO stimulation in comparison to the basal level prior to CNO stimulation. We observed a significant increase in intracellular Ca^2+^ upon CNO stimulation in hM_3_D expressing cells (p < 0.0001). We also excluded receptor desensitization or internalization by repetitive Ca^2+^ imaging experiments at 0 hrs and 24 hrs after the initial stimulation (Fig. 1F). This data clearly demonstrates the functionality of the hM_3_D-receptor and the ability to induce an increase of intracellular calcium levels *in vitro*.

### Increased intracellular Ca^2+^ levels after CNO stimulation cause Rac1 activation

**Figure 2:**
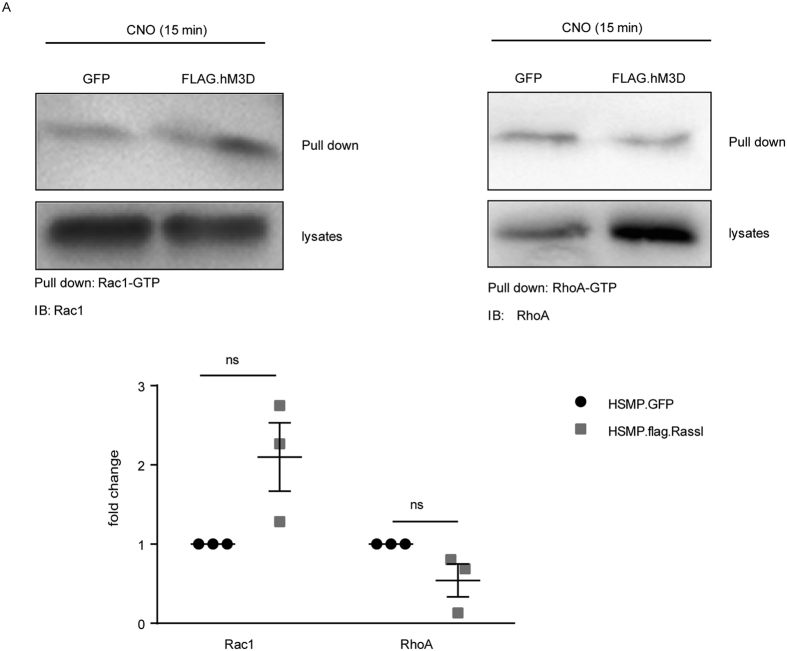
Increased intracellular Ca^2+^-levels after CNO treatment cause Rac1 activation. (**A**) GTPase pull-down assays were performed for active RhoA and Rac1. FLAG.hM_3_D expressing podocytes and GFP expressing control podocytes were stimulated with 1 μM CNO for 15 min. Pull down of active Rac1 and RhoA revealed no significant changes of active Rac1 or RhoA in FLAG.hM_3_D-expressing cells in comparison to control cells. Active Rac1 and RhoA levels were normalized to tubulin. (two-tailed t-test: p > 0.05).

Small GTPases are known to be crucial for podocyte function and maintenance^21^,^22^ and are under tight control of intracellular Ca^2+^ levels^27^. Moreover, GTPases play a crucial role for the organisation of the actin cytoskeleton and cell migration^28^. To investigate the effects of increased intracellular Ca^2+^ levels after CNO stimulation on small GTPases in podocytes, we performed GTPase pull-down assays for active Rac1 and RhoA. Podocytes were treated with 1 μM CNO for 15 min and the GTP-bound active Rac1 and RhoA were pulled down (Fig. 2A). Comparing the GFP-expressing control cells with the FLAG.hM_3_D-expressing podocytes revealed no statistically significant differences of active Rac1 or RhoA levels in the receptor expressing cells in comparison to control cells, although a trend towards increased active Rac1 and decreased RhoA levels could be observed (Fig. 2A).

### Generation of a transgenic podocyte-specific hM_3_D mouse model

**Figure 3:**
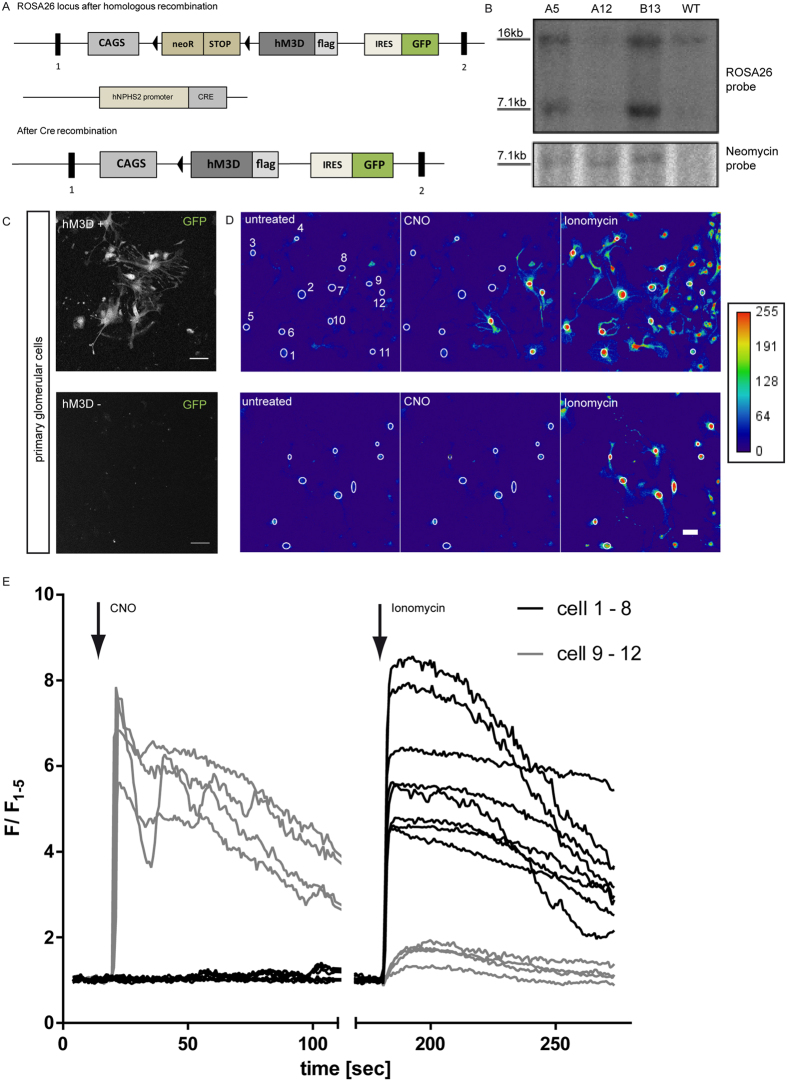
Generation of a transgenic podocyte-specific hM_3_D mouse model. (**A**) Schematic of the ROSA26 locus with the FLAG.hM_3_D sequence before and after Cre recombination. Triangles indicate loxP sites and rectangles indicate exons. (**B**) Southern Blot analyses were performed with a ROSA26 and a neomycin probe to screen for positive ES-cell clones. (**C**) Isolated primary glomerular cells were cultured and GFP expression was visualized by confocal microscopy. Glomerular cells from ROSA26^hM3D/wt^; PodCre^tg/wt^ show cytoplasmic GFP expression. ROSA26^wt/wt^; PodCre^tg/wt^ control cells were GFP negative. (scale bar = 50 μm). (**D,E**) Representative images of Ca^2+^ imaging with Fluo-8 (scale bar = 20 μm). Primary glomerular cells were used for Ca^2+^ imaging with Fluo-8. Cells expressing the receptor showed an instant increase of intracellular Ca^2+^ levels after administration of 100 μM CNO. Control cells did not react to CNO (scale bar = 20 μm). Experiments were performed in three biological replicates. Fluorescence is depicted as F/F_1–5_, where the mean value of the first five measurements prior to CNO stimulation was used for normalisation.

Next, we generated a knock-in mouse model to manipulate Ca^2+^ levels in podocytes specifically *in vivo*. To this end, a ROSA26 targeting vector was generated in which expression of the C-terminal FLAG-tagged hM_3_D construct is driven by a *CAGS* promotor as published previously^29^. To ascertain cell type specific expression of the receptor transgene a loxP-flanked Neo.Stop cassette was inserted upstream of the receptor sequence. In addition, we included an IRES.GFP to visualize transgene expression (Fig. 3A). C57/BL6 ES cells of the Bruce 4 lineage were transfected with the targeting vector and positive clones were identified via selection and southern blot analysis (Fig. 3B). Positive clones were used to generate chimeras that were intercrossed with C57/BL6 mice to establish the ROSA26 LoxP-STOP-LoxP hM_3_D mouse strain. Mice carrying the hM_3_D construct in the ROSA26 locus were mated with Nphs2. Cre mice to achieve a podocyte specific receptor expression after Cre mediated recombination and deletion of the STOP codon (Fig. 3A)^30^. We further validated expression of the receptor by isolating and culturing primary glomerular cells *in vitro*. Podocytes from hM_3_D-transgenic mice also expressed GFP and could easily been identified (Fig. 3C). To validate the functionality of the hM_3_D receptor in primary podocytes we labelled the cells with the Ca^2+^ indicator dye Fluo-8 and stimulated the cells with 100 μM CNO. hM_3_D/GFP-positive cells showed a strong increase in the intracellular Ca^2+^-levels upon CNO treatment, while hM_3_D-negative cells did not respond to CNO treatment (Fig. 3D). Experiments were performed in three biological replicates. Analyses of the signal intensities revealed an immediate increase of intracellular Ca^2+^ with a constant decline of the signal (Fig. 3E). Of note, the strong oscillatory phenotype observed in some virus transduced cultured podocytes was not seen in primary cells. Other glomerular cells, like endothelial or mesangial cells, which were co-cultured in this mixed primary cell culture system, served as internal controls and did not respond to CNO nor did the outgrowing glomerular cells from hM_3_D negative control animals. Thus, our data clearly shows the podocyte-specific expression of the hM_3_D receptor and its functionality in isolated primary podocytes.

### Administration of CNO leads to increased Ca^2+^-levels in podocytes *in vivo*

**Figure 4:**
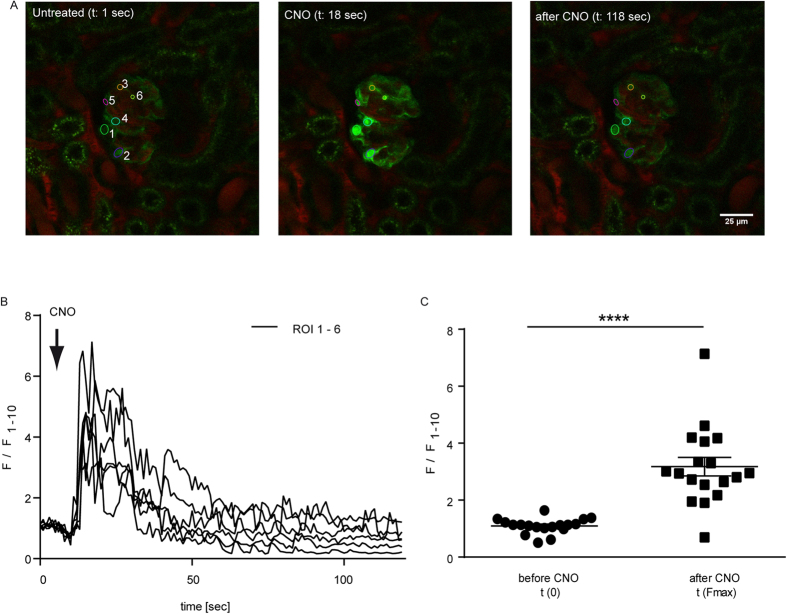
Administration of CNO leads to increased Ca^2+^-levels in podocytes *in vivo.* (**A**) Glomeruli of ROSA26^hM3D/wt^; NPHS2.Cre^tg/wt^; GCaMP3^tg/wt^ mice were visualised in the intact kidney *in vivo* using a Multi-Photon-microscope. CNO (5 mg/kg) was injected i.a. after 10  sec and caused an immediate increase in intracellular Ca^2+^ levels (scale bar = 25 μm). (**B**) Fluorescence is depicted as F/F_1–10_, where the mean value of the first ten measurements (F_1–10_) prior to CNO stimulation was used for normalisation. Experiments were performed in three biological replicates. (**C**) Statistical analysis performed for Multi-Photon-imaging experiments revealed a significant increase of the intracellular Ca^2+^ after CNO stimulation. N = 18 cells were included in the statistical analysis (t-test p < 0.0001).

To assess the functionality of the receptor *in vivo* we mated our ROSA^hM3D/wt^; Nphs2.Cre^tg/wt^ mice with the Ca^2+^-reporter strain GCaMP3^tg/wt^ 31. Using Multi-Photon-microscopy we were able to study the effects of CNO administration on podocytes *in vivo*. After i.a. injection of 5 mg/kg bodyweight CNO an immediate elevation of the intracellular Ca^2+^ levels was observed in podocytes (Fig. 4A,B). All podocytes responded simultaneously and showed a significant single transient Ca^2+^ peak (Fig. 4B,C).

### hM_3_D-mediated Ca^2+^-peaks do not effect glomerular filtration or *ex vivo* kidney perfusion

**Figure 5:**
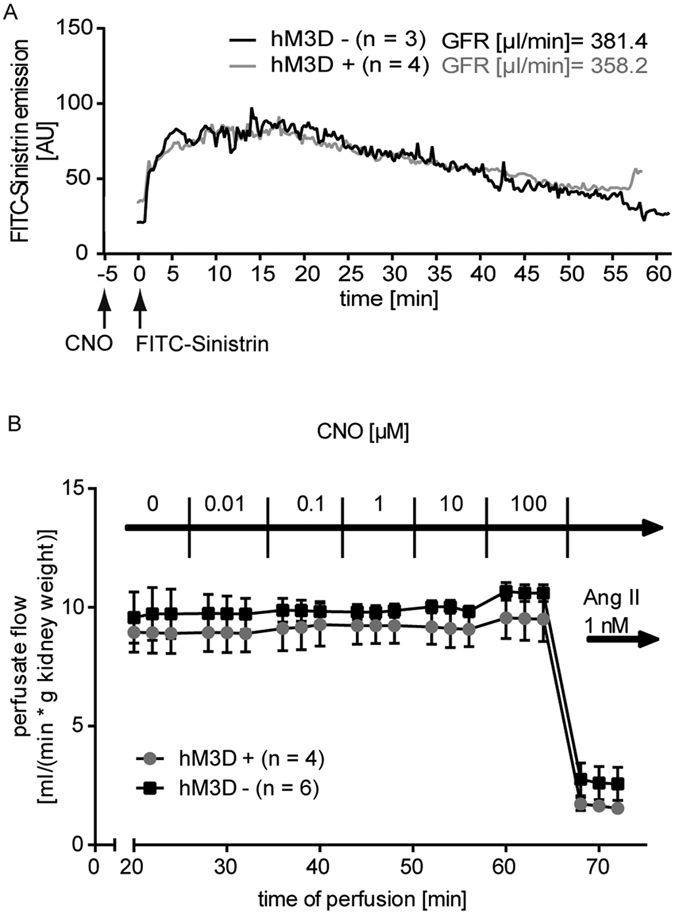
Increased intracellular Ca^2+^levels do not affect glomerular filtration or *ex vivo* kidney perfusion. (**A**) Transcutaneous GFR measurement with hM_3_D-expressing animals in comparison to their littermate controls does not reveal changes after CNO (5 mg/kg bodyweight) administration. Displayed are means (n = 4: hM3D positive, n = 3: hM3D negative). (**B**) *Ex vivo* kidney perfusion was performed with increasing CNO concentrations. Comparing hM_3_D-expressing mice with their control littermates does not show differences in the vascular resistance. Angiotensin II was used as positive control. Displayed are means with standard error of the mean (SEM).

Having shown the feasibility of our transgenic mouse model to transiently increase Ca^2+^ levels in podocytes we set out to study the effects of CNO and a subsequent increase of intracellular Ca^2+^-levels in podocytes on glomerular filtration and glomerular perfusion. First, we performed transcutaneous glomerular filtration rate measurements to study the glomerular filtration rate (GFR) as published by Schreiber *et al*.^32^. Using this method, we were able to record the glomerular filtration rate after CNO administration in freely moving mice over 60 minutes. We did not observe any significant differences in GFR between hM_3_D-expressing animals in comparison to their littermate controls (Fig. 5A).

Second, we investigated the impact of increased intracellular Ca^2+^-levels on glomerular perfusion. As podocytes harbour a contractile actin-myosin-based machinery^33^ we hypothesized, that podocytes adapt their contractile behaviour due to the changes in Ca^2+^ concentration, which in turn might alter vascular resistance. To this end, we performed *ex vivo* perfusion experiments as published previously^34^. Kidneys of hM_3_D-expressing mice and their littermate controls were subjected with increasing CNO concentrations, starting at 0.01 μM up to 100 μM. However, we could not observe a change in the vascular resistance after CNO administration (Fig. 5B). Administration of angiotensin II, which leads to constriction of the vas afferens, resulted in an immediate and strong decrease of the perfusion rate and served as positive control. Taken together, these results show that administration of CNO and a subsequent increase in intracellular Ca^2+^ does not change the glomerular filtration rate nor renal vascular resistance.

### Increased intracellular Ca^2+^-levels do not cause glomerular disease

**Figure 6:**
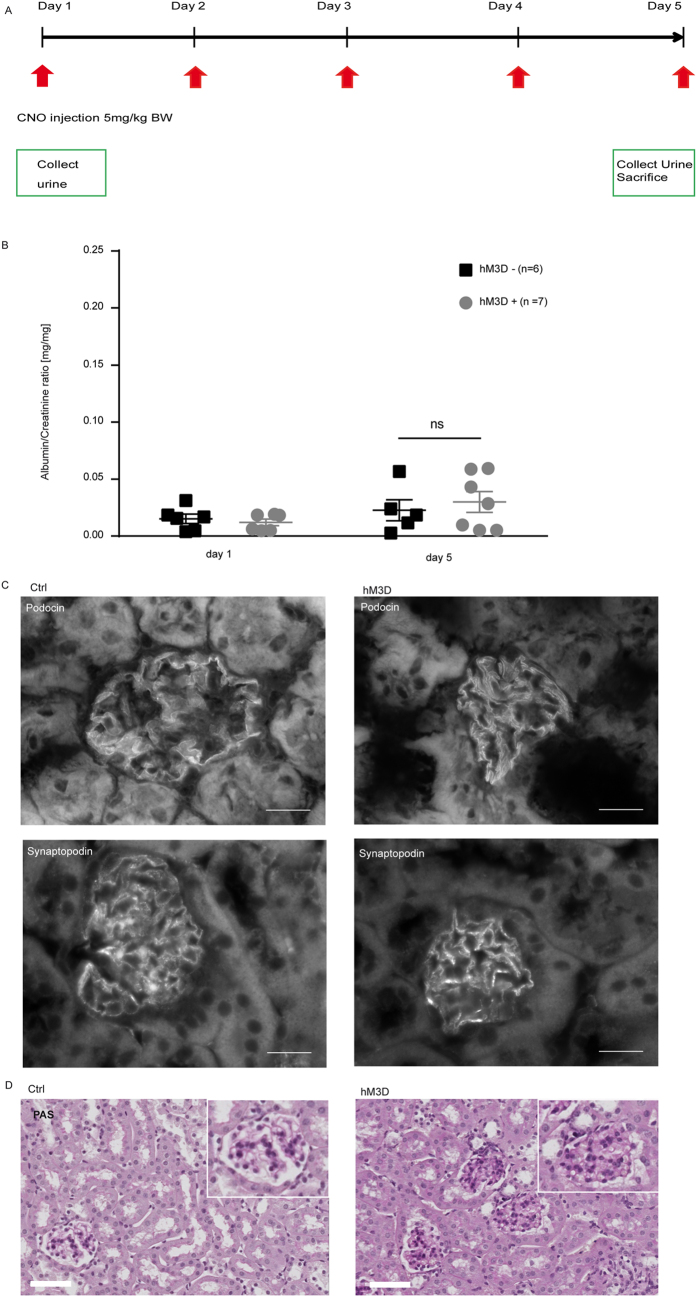
Increased intracellular Ca^2+^-levels do not cause glomerular disease. (**A**) Scheme of CNO administration. 5 mg/kg bodyweight CNO was injected i.p. on 5 consecutive days. (**B**) Urinary albumin to creatinine ratios did not show an increase after 5 days of CNO administration in both groups. Displayed are means with SEM; 2way ANOVA and Bonferroni’s multiple comparison test (p > 0.05). (**C**) Kidney sections of hM_3_D-expressing animals and their littermate controls after 5 days of CNO treatment were stained with a podocin and synaptopodin antibody to visualize the slit diaphragm and the actin cytoskeleton respectively (scale bar = 20 μm). (**D**) PAS (periodic acid Schiff stainings) were performed on day 5 of CNO administration and did not reveal differences in histology (scale bar = 50 μm).

**Figure 7:**
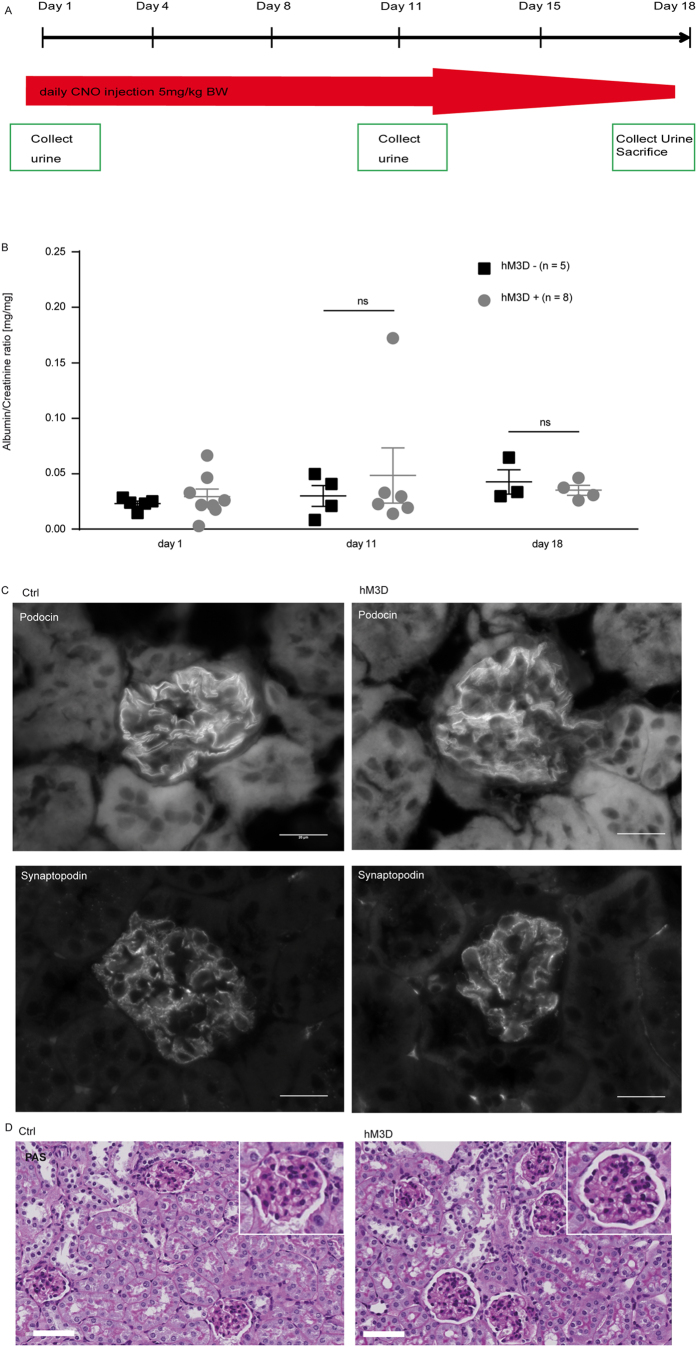
Long-term CNO treatment does not cause glomerular disease. (**A**) Scheme of CNO administration. 5 mg/kg bodyweight CNO was injected i.p. on 18 consecutive days. (**B**) Urinary albumin to creatinine ratios did not show an increase after 18 days of CNO administration in both groups. Displayed are means with standard error of the mean (SEM); 2 way ANOVA and Bonferroni’s multiple comparison test (p > 0.05). (**C**) Kidney sections of hM_3_D-expressing animals and their littermate controls after 18 days of CNO stimulation were stained with a podocin and synaptopodin antibody to visualize the slit diaphragm and the actin cytoskeleton respectively (scale bar = 20 μm). (**D**) PAS (periodic-acid Schiff stainings) were performed on day 18 of CNO administration and did not reveal differences in histology (scale bar = 50 μm).

After investigating short-term effects of increased intracellular Ca^2+^ levels on podocyte function, we next investigated effects after repetitive administration of CNO. Animals expressing the hM_3_D construct and their littermate controls received CNO for 5 consecutive days (5 mg/kg bodyweight i.p.). However, we could not observe any signs of glomerular disease based on histological analyses as well as urinary albumin-to-creatinine ratios (Fig. 6B–D). Immunofluorescence stainings for the actin-binding protein synaptopodin as well as the slit diaphragm protein podocin did also not reveal differences between the hM_3_D- expressing and control mice (Fig. 6C). Next, to investigate if a prolonged application of CNO causes glomerular disease, we administered CNO (5 mg/kg bodyweight i.p.) to mice expressing the hM_3_D construct and their control hM_3_D-negative littermates for 18 consecutive days. Again, urinary and histological analyses did not reveal any signs of glomerular disease (Fig. 7B–D). In conclusion, even a repetitive administration of CNO for 18 days failed to induce glomerular disease.

## Discussion

In this study, we present a novel transgenic mouse model to study Ca^2+^ signalling in podocytes *in vivo*. We used a modified *DREADD*, a Gq-coupled human muscarinic type 3 receptor (hM_3_D). Due to two point mutations (Y149C/A239G) this receptor can solely be activated by the chemical compound CNO but no longer by its native ligand acetylcholine^25^,^26^. Moreover, great advantages of the usage of CNO are its bioavailability for rodents^35^, the natural affinity of its parent compound clozapine to M_3_ receptors, which makes CNO a potent agonist of the mutated M_3_ receptor^26^ and its pharmacological inert behaviour^25^,^36^. The highly specific binding of CNO to the receptor causes activation of the Phospholipase C (PLC) pathway in the targeted cell type. This activation leads to binding of IP_3_ to the IP_3_-receptor at the ER resulting in a strong Ca^2+^ release from the ER into the cytoplasm. This allows studying effects of increasing intracellular Ca^2+^ levels *in vivo* in a novel, highly specific manner. As we inserted a LoxP-STOP-LoxP cassette in the ROSA26 locus upstream of the hM_3_D sequence, this mouse model may be used to study effects of Gq-coupled receptor activation in a variety of tissues and cell types. Here, we mated the ROSA^hM3D/wt^ mice to a podocyte-specific Cre recombinase driver line (NPHS2.Cre^tg/wt^)^30^ to achieve exclusive expression of the receptor transgene in podocytes. Doing so, the increase of intracellular Ca^2+^ after CNO administration solely occurred in podocytes.

Surprisingly, despite the proven functionality of the hM_3_D receptor in podocytes as demonstrated *in vitro*, *ex vivo* and *in vivo*, activation of the PLC pathway did not affect glomerular filtration or perfusion nor caused glomerular disease even after a prolonged stimulation for 18 consecutive days. Apparently, short and transient Ca^2+^ peaks as visualized by calcium imaging *in vivo* in this study are not sufficient to cause overt morphological alterations or functional changes at the renal filtration barrier. We did not observe any effect on glomerular perfusion, filtration or the onset of albuminuria. Our findings are in line with previously reported effects of G-protein coupled receptors in regulating glomerular biology. Expression of a constitutively active Gq-coupled receptor mutant in podocytes did also not result in any glomerular dysfunction under physiological conditions^37^. Only when a second hit was present, e.g. the PAN nephrosis model or diabetic conditions, glomerular malfunction was aggravated to a greater extent as compared to wildtype controls^37^.

These findings do not allow the assumption that Ca^2+^ signalling events are dispensable for podocyte function. As we activated the PLC pathway, increased Ca^2+^ levels primarily derive from the ER. This might be of particular importance in podocytes with their delicate cytoskeletal architecture. Increased Ca^2+^ levels close to the ER, which is primarily found in perinuclear regions in the podocyte cell body, might not resemble the intracellular Ca^2+^ concentration as observed in patients with TRPC6 gain-of-function mutations^8^. Moreover, podocytes rely on a stringent control of their intracellular Ca^2+^ levels as they harbor several homeostasis mechanisms including the Na^+^-Ca^2+^-exchanger, the ATP-dependent plasma membrane Ca^2+^ pump (PMCA) as well as Ca^2+^-buffers like calbindin and parvalbumin^38^. It is well conceivable that increasing intracellular Ca^2+^ levels due to the CNO administration might be compensated by these homeostasis mechanisms. In addition, the slit diaphragm might serve as a Ca^2+^ microdomain orchestrating Ca^2+^-dependent proteins like the TRPC channels, GTPases and synaptopodin. Furthermore, administration of CNO did only result in a transient Ca^2+^ peak in podocytes.

One might also speculate, whether the hM_3_D-transgene gets rapidly internalized or undergoes desensitization upon stimulation. However, studies performed in hippocampal neurons expressing a similar hM_3_D-transgene showed repetitive activation of the receptor 24 hrs after the initial CNO treatment^25^, suggesting that receptor internalisation and desensitization does not occur. Similarly, we could observe a repetitive activation of the receptor and subsequent increase of intracellular Ca^2+^ levels by stimulating podocytes with CNO at 0 and 24 hrs. It is therefore easily conceivable, that a sustained Ca^2+^ influx rather than a single Ca^2+^ peak is required to induce cytoskeletal changes to cause foot process effacement in podocytes.

In conclusion, we present a novel *DREADD* mouse model to study Ca^2+^ signalling events *in vivo*. As the *DREADD* sequence is integrated into the ROSA26 locus it can be easily applied for all murine tissues by mating the loxP-STOP-loxP hM_3_D mice with tissue specific Cre recombinase mouse lines. In podocytes, a transient Ca^2+^ peak alone is not sufficient to impair podocyte function challenging the current view of transient Ca^2+^ peak as regulators of glomerular biology.

## Material and Methods

### Cell culture

Immortalized mouse podocytes were cultured with RPMI-1640 medium (Sigma-Aldrich, St. Louis, USA) with 10% fetal bovine serum, 5% sodium pyruvate solution 100 mM (Sigma-Aldrich, St. Louis, USA) and 5% HEPES buffer solution 1 M (Life Technologies, Carlsbad, USA) as previously reported^39^. The undifferentiated and proliferating cells were cultured at 33 °C in the presence of 2.5 μl murine IFN-gamma (Provitro, Berlin, Germany). To induce podocyte differentiation the cells were shifted to 37 °C for 14 days in the absence of IFN-gamma. To generate podocyte cell lines with stable expression of FLAG-tagged hM_3_D or GFP we used the commercially available pLenti6/V5-dest vector (Thermo Fisher, Waltham, USA) as previously published^40^. To this end, podocytes were grown at 33 °C and transduced with a Lentivirus containing the hM_3_D or GFP construct respectively.

### Immunofluorescence on cells

Immortalized mouse podocytes were grown at 37 °C for 14 days. The cells were fixed with 4% paraformaldehyde, further blocking was performed with 5% normal donkey serum for 30 min. The cells were washed with 1 x PBS (phosphate buffered saline: 137 mM NaCl, 2.7 mM KCl, 10 mM Na_2_HPO_4_ and 2 mM KH_2_PO_4_) containing 1 mM CaCl_2_ and 0.5 mM MgCl_2_ for three times. The cells were stained with an anti-FLAG antibody over night at 4 °C. The primary antibody was diluted in 1 x PBS with 1 mM CaCl_2_, 0.5 mM MgCl_2_ and 0.1% Triton-X and with 5% NDS. Prior to incubation with the secondary antibody, cells were washed again three times with 1 x PBS with 1 mM CaCl_2_ and 0.5 mM MgCl_2_. The respective secondary antibody was diluted in 1 x PBS with 1 mM CaCl_2_, 0.5 mM MgCl_2_ and 0.1% Triton-X. Mounting was performed with Prolong Gold with DAPI (Thermo Fisher, Waltham, USA). Antibody dilution and commercial source are listed in Table 1. All images were taken with an Axiovert 200 M microscope/C-Apochromat 63x/1.20 W water immersion objective (all from Carl Zeiss MicroImaging GmbH). Images were further processed with ImageJ/Fiji Software and Photoshop CS4 version 11.0.

### Western Blot

Immortalized mouse podocytes were stimulated with 1 μM CNO (Enzo Life Sciences, Farmingdale, USA) diluted in medium for 10 min. Afterwards the cells were harvested using a cell scraper and lysed in 100 μl 1% Trition-X 100 buffer (20 mM Tris-HCl pH 7.5, 50 mM NaCl, 50 mM NaF, 15 mM Na_4_P_2_O_7_) and 1 mM of the serine protease inhibitor PMSF Phenylmethanesulfonyl fluoride (Sigma-Aldrich, St. Louis, USA) and 2 mM of the phosphatase inhibitor Na_3_VO_4_ (Sigma-Aldrich, St. Louis, USA). Following incubation on ice for 15 min and subsequent centrifugation (14.000 × g, 4 °C, 15 min) the supernatant was used for further analyses. The diluted lysates (with 2x SDS-PAGE buffer) were separated by SDS-PAGE. For gel electrophoresis the XCell SureLockTM Mini-Cell System was used. Electrophoresis was performed with 70 V for 30 min, followed by 25 mA/gel for 1:45 h. Blotting was performed on PVDF membranes (Carl Roth, Karlsruhe, Germany) for 1 h at 12 V. After the transfer, membranes were blocked with 5% bovine serum albumin (BSA) (Sigma-Aldrich, St. Louis, USA) for 30 min, followed by three washing steps. The primary antibody was diluted in washing buffer (30 mM Tris, 300 mM NaCl and 0.3% (v/v) Tween20, pH 7.5) and incubation was performed over night at 4 °C. The respective secondary antibody was diluted in washing buffer and incubated 45 min at room temperature, followed by visualization with chemiluminescence with a Fusion Solo S (Vilber Lourmat, Eberhardzell, Germany). Antibody dilution and commercial source are listed in Table 1.

### Ca^2+^-imaging

Immortalized mouse podocytes were grown at 33 °C and cultured on 8 chamber slides (ibidi GmbH, Planegg/Martinsried, Germany). The cells were incubated with 5 μM Fluo-8 (Sigma-Aldrich, St. Louis, USA) in imaging buffer (125 mM NaCl, 5 mM KCl, 1.2 mM MgSO_4_*7H_2_O, 25 mM HEPES, 6 mM Glucose) for 30 min at 37 °C. Afterwards they were kept in imaging buffer for 20 min at room temperature. Imaging was performed with a Confocal microscope LSM710/Axiobserver Z1(Carl Zeiss Microimaging, Jena, Germany) using an 40x objective. Pictures were taken every 0.78 secs. 2 μM CNO was added after 10 pictures for the immortalized mouse podocytes and 100 μM after 20 pictures for the primary glomerular cells. 3 μM Ionomycin was used as positive control and added to the immortalized cells after 280 pictures and after 130 pictures to the primary cells.

### GTPase pull down assay

Immortalized mouse podocytes were grown at 33 °C. Five confluent dishes of each cell line were stimulated with 1 μM CNO. After 15 min the cells were pooled and lysed according to the according to manufacturer’s instructions. Rho and Rac1 pull down assays (Thermo Fisher, Waltham, USA) were performed according to manufacturer’s instructions.

### Transgenic mouse models

Generation of the ROSA26^hM3D/wt^ mice is described in the results section in detail. Briefly, to generate mice expressing the hM_3_D transgene in podocytes, we mated ROSA26^hM3D/wt^ mice to NPHS2.Cre^tg/wt^ mice^30^. Multi-Photon-imaging was performed using the Ca^2+^ reporter mouse GCaMP3^tg/wt^ 31. ROSA^hM3D/wt^; NPHS2.Cre^tg/wt^ mice were mated with GCaMP3^tg/wt^ to achieve a simultaneous hM_3_D and Ca^2+^ reporter expression in podocytes exclusively. All animals were on a pure C57/Bl6 background. The mouse holding was done in the University of Cologne animal facility according to standardized specific pathogen-free conditions. All animal experiments were performed in accordance with the guidelines provided by the LANUV NRW (Landesamt für Natur, Umwelt und Verbraucherschutz Nordrhein-Westfalen/State Agency for Nature, Environment and Consumer Protection North Rhine-Westphalia). The experimental protocol was examined and approved by the LANUV NRW.

### Isolation of primary cells

To isolate primary cells we perfused the kidneys via the renal arteries with magnetic beads (Invitrogen, Carlsbad, USA) and isolated glomeruli as previously published^41^,^42^. The isolated glomeruli were transferred in 12-well dishes with RPMI-1640 medium (Sigma-Aldrich, St. Louis, USA) with 10% fetal bovine serum, 5% sodium pyruvate solution 100 mM (Sigma-Aldrich, St. Louis, USA) and 5% HEPES buffer solution 1 M (Life Technologies, Carlsbad, USA). After three days glomeruli were detached from the surface with ice cold PBS. All cells grown on the dish remained attached and were used for further analysis.

### Administration of CNO *in vivo*

Mice were injected intra-peritoneally with 5 mg/kg bodyweight CNO (Enzo Life Sciences, Farmingdale, USA) dissolved in NaCl either 5 or 18 consecutive days as published previously^25^. Spot urine was collected on day 1 and day 5 and day 1, day 11 and day 18 respectively. Tissue samples were harvested at day of sacrifice.

### Urinary analysis

Quantification of the urinary albumin levels was performed with a specific mouse albumin ELISA kit according to the manufactor’s instructions (ICL/Dunn Labortechnik GmbH, Asbach, Germany). Creatinine levels were quantified with a urinary creatinine kit according to the manufactor’s instructions (Biomol, Hamburg, Germany).

### Histological analysis on mouse tissue

Mice were perfused with phosphate-buffered saline via the heart and kidneys were either fixed in 4% paraformaldehyde overnight or frozen in Tissue-Tek^®^ O.C.T.™ compound (Sakura, Leiden, Netherlands). Paraformaldehyde fixed kidneys were embedded in paraffin and used for cutting 2 μm thick sections. To study morphological changes we performed a periodic-acid Schiff staining. To this end, sections were stained with 0.9% periodic acid (Carl Roth, Karlsruhe, Germany) for 10 min, followed by incubation in Schiff’s reagent for 10 min (VWR, Radnor, USA). After washing with tap water for 2 min sections were incubated for 10 min in Mayer’s hematoxylin solution (Sigma-Aldrich, St. Louis, USA). Mounting was performed with histomount. For immunofluorescence analysis frozen kidney sections (5 μm) were used. After fixation with paraformaldehyde sections were blocked with 5% normal donkey serum in phosphate buffered saline with 0, 1% Triton-X (PBS-T) and subsequently incubated with primary antibody diluted in PBS-T with 5% NDS overnight at 4 °C. The respective secondary antibody was diluted in PBS-T and incubation was performed for 45 min at room temperature. Mounting was done with Prolong Gold antifade with DAPI (Thermo Fisher, Waltham, USA). All images were taken with an Axiovert 200 M microscope/C-Apochromat 63x/1.20 W (all from Carl Zeiss MicroImaging GmbH, Jena, Germany). Images were further processed with ImageJ/Fiji Software and Photoshop CS4 version 11.0. Antibody dilution and commercial source are listed in Table 1.

### Multi-Photon – Calcium imaging

4 weeks old ROSA^hM3D/wt^; NPHS2.Cre^tg/wt^; GCaMP3^tg/wt^ mice were anaesthetized using isoflurane and buprenorphine (0.1 mg/kg). A tube was placed into the trachea to facilitate breathing, and the right carotid artery was cannulated for dye infusion and injection of CNO. 70 kDa Texas red dextrane was injected i.a. to label the vasculature. The left kidney was exteriorized via a small incision at the left flank of the animal. The mouse was placed onto a heated mouse holder and the kidney was stabilized and covered with a coverslip for imaging. Body temperature was maintained during the imaging time. Calcium imaging was performed with a TSC SP8 upright Multi-Photon microscope (Leica) using an IR Apo L25x/0.95 W objective and a Coherent Chameleon Vision II laser at a wavelength of 940 nm. For acquisition two external hybrid detectors were used. The time courses of calcium signals were acquired at 1 frame per second over 90 seconds. After 10 seconds of baseline recording, 5 mg/kg bodyweight CNO was injected. The images were further processed with Leica Application Suite X, Version 1.1.0.

### Transcutaneous glomerular filtration rate measurement

To study the glomerular filtration rate we made use of a transcutaneous measurement approach^32^. Therefore ROSA^hM3D/wt^; NPHS2.Cre^tg/wt^ mice were anaesthetized with isofluran and a sensor was taped directly on their skin. After injecting 5 mg/kg CNO i.p., we injected 15 mg/100 g FITC-Sinistrin i.v. and measured the GFR for 60 min in the freely moving mice. Calculation of the GFR was performed according to Schreiber *et al*.^32^.

### Isolated perfused mouse kidney

Kidneys from ROSA^hM3D/wt^; NPHS2.Cre^tg/wt^ mice and their littermate controls were perfused *ex-situ* at a constant perfusion pressure (100 mmHg) as described in detail previously^34^. Perfusion medium consisted of a modified Krebs-Henseleit buffer supplemented with bovine serum albumin (6 g/100 ml) and human erythrocytes (10% hematocrit). The renal vein was cannulated and samples of the venous perfusate were collected and weighed every 2 minutes for the determination of renal blood flow. Three samples were taken during each experimental period and the last two values were averaged for statistical analysis.

### Statistical analysis

All results are expressed as means ± SEM, except for the GFR measurement, which is depicted as mean. Statistical significance was evaluated using GraphPad Prism version 6 for Windows (GraphPad Software, San Diego, CA). For two groups t-test was applied and a *P*-value < 0.05 was considered significant. For two groups with two independent variables 2 way ANOVA combined with Bonferroni’s multiple comparison test was applied and a *P*-value < 0.05 was considered significant.

## Additional Information

**How to cite this article**: Koehler, S. *et al*. Single and Transient Ca^2+^ Peaks in Podocytes do not induce Changes in Glomerular Filtration and Perfusion. *Sci. Rep.* **6**, 35400; doi: 10.1038/srep35400 (2016).

## Figures and Tables

**Table 1:** Antibodies.

Name	Company	Catalog No.	host species	Dilution IF	Dilution WB
Anti-FLAG	Sigma	F7425	Rabbit	1:100	
Anti-Podocin	Sigma	P0372	Rabbit	1:100	
Anti-pMEK1/2	Cell signaling	2338S	Rabbit		1:1000
Anti-MEK1/2	Cell signaling	9126	Rabbit		1:1000
Anti-Synaptopodin	PROGEN Biotechnik	65294	mouse	1:10	
goat anti-rabbit IgG HRP conjugated	Jackson Immuno Research	111-035-003	Goat		1:30.000
donkey anti-rabbit IgG (H + L)-Alexa Fluo 488	Jackson Immuno Research	711-545-152	Donkey	1:250	
donkey anti-rabbit IgG (H + L)-Cy3, MinX	Jackson Immuno Research	711-165-152	Donkey	1:250	
